# Correlation between endo-perio lesions and systemic diseases: A cross-sectional study

**DOI:** 10.6026/973206300211144

**Published:** 2025-05-31

**Authors:** Rahul Singh, Nidhi Singh, Simran Chauhan, Lubna Tabassum Siddiqui, Lucy Bhola, Madhuri S. Sale, Vinayak Meharwade, Prabu Mahin Syed Ismail, Abhigyan Manas

**Affiliations:** 1 Department of Conservative Dentistry and Endodontics, Sarjug Dental College and Hospital, Darbanga, India; 2 Department of Dentistry, Government Medical College, Pali, Rajasthan, India; 3 Department of Health Informatics, Dental Surgeon, Himachal University, Maharaja Nagar, Ludhiana-141001, Punjab, India; 4 Department of Periodontics, Swargiya Dadasaheb Kalmegh Smruti Dental College and Hospital, Wanadongri, Hingna, Nagpur, Maharashtra, India; 5 Department of Periodontics and Oral Implantology, Kalinga Institute of Dental Sciences (KIDS), KIIT Deemed to be University, Bhubaneswar, Odisha, 751024, India; 6 Department of Oral Pathology and Microbiology, Bharati Vidyapeeth (Deemed to be University) Dental College & Hospital, Sangli, Maharashtra, India; 7 Department of Periodontology, Sinhgad Dental College and Hospital, Pune, Maharashtra, India; 8 Department of Conservative Dentistry and Endodontics, College of Dentistry, Qassim University, Kingdom of Saudi Arabia; 9 Department of Oral and Maxillofacial Surgery, Faculty of Dental Sciences, Uttar Pradesh University of Medical Sciences, Saifai, Etawah, India

**Keywords:** Endo-perio lesions, systemic diseases, diabetes mellitus, cardiovascular disorders, periodontal-endodontic correlation, age and gender association, oral-systemic link

## Abstract

The correlation between endo-perio lesions and systemic diseases and to analyze their association with age and gender distribution is of interest. Hence, a total of 800 participants aged between 25 and 75 years. Patients were categorized based on the presence or absence of systemic diseases, including diabetes, hypertension, cardiovascular disorders, arthritis and altered kidney/liver function. Detailed clinical and radiographic evaluations were conducted to classify lesions as primary endodontic, primary periodontal, or true combined lesions. The study underscores a strong correlation between systemic diseases and the severity of endo-perio lesions, especially in older males.

## Background:

Endodontic-periodontal (endo-perio) lesions are complex pathological entities involving the pulp and periodontal tissues, often presenting significant diagnostic and therapeutic challenges in dental practice [1]. These lesions may develop from a primary pulpal infection extending to the periodontium, a primary periodontal disease affecting the pulp, or true combined lesions where both tissues are simultaneously compromised [2]. Growing evidence the impact of systemic health conditions-such as diabetes mellitus, hypertension, cardiovascular diseases, liver dysfunction, arthritis and renal impairment-on the initiation, progression and resolution of endo-perio lesions [3]. For instance, diabetes mellitus leads to impaired neutrophil function and increased production of pro-inflammatory cytokines, contributing to delayed healing and heightened tissue destruction [4]. Hypertension may reduce periodontal flow, affecting nutrient supply and immune surveillance [5]. Cardiovascular diseases are often associated with systemic inflammation and altered vascular responses, which can exacerbate local periodontal inflammation [6]. Liver disorders may impair clotting factors and metabolic clearance, influencing wound healing, while kidney dysfunction can alter bone metabolism and immune regulation [7]. Autoimmune conditions like arthritis contribute to chronic inflammation that may enhance periodontal breakdown and hinder tissue repair [8]. Therefore, it is of interest to evaluate the correlation between endo-perio lesions and systemic diseases, and to analyze how age and gender influence the prevalence and pattern of these lesions.

## Materials and Methods:

This cross-sectional observational study was conducted over a six-month period in the Department of Conservative Dentistry and Periodontology at a tertiary care dental institution. Ethical clearance was obtained from the institutional review board before the study began. A total of 800 participants aged 25 to 75 years were enrolled using a convenience sampling method. Informed consent was obtained from all subjects prior to clinical examination and data collection. The inclusion criteria for the study were patients aged between 25 and 75 years, with a diagnosis of endodontic, periodontal, or combined endo-perio lesions. Patients with or without documented systemic diseases, such as diabetes mellitus, hypertension, cardiovascular disease, arthritis, liver dysfunction, or altered kidney function, were eligible for inclusion. Patients undergoing active immunosuppressive therapy or chemotherapy, as well as pregnant or lactating women, were excluded from the study. Additionally, subjects with incomplete medical histories or unwillingness to participate were excluded. Clinical evaluations were conducted for all participants, including periodontal probing, percussion sensitivity, mobility assessment and pulp vitality testing. Radiographic assessments were carried out using periapical radiographs and orthopantomograms to evaluate lesion extent and bone involvement. Based on clinical and radiographic findings, patients were categorized into three groups: primary endodontic lesion, primary periodontal lesion, and true combined lesion. Systemic conditions were confirmed through detailed medical history, physician reports, and, when necessary, laboratory reports, including fasting blood sugar, blood pressure readings and liver and renal function tests. Demographic data, including age and gender, along with the dental diagnosis and systemic disease status, were recorded in a structured data collection form. Participants were grouped into three age categories: 25-40 years, 41-55 years and 56-75 years. Statistical analysis was performed using SPSS version 25.0. Descriptive statistics were applied to determine frequencies and percentages. The chi-square test was used to examine associations between lesion types and systemic diseases. Pearson's correlation coefficient was used to assess the relationship between age, gender, lesion severity and systemic involvement, with a p-value of less than 0.05 considered statistically significant.

## Results:

The study examined the demographic distribution, prevalence of systemic diseases and the association of systemic diseases with -perio lesions among a cohort of 800 participants. Table 1 shows demographic distribution of participants. The gender distribution among the participants revealed a slight male dominance, with 52.5% (420) male and 47.5% (380) female participants. Age-wise, the cohort was predominantly distributed between the age groups of 41-55 years (41.25%, 330 participants) and 56-75 years (36.25%, 290 participants), followed by the 25-40 years age group, which accounted for 22.5% (180 participants). Table shows prevalence of Systemic Diseases. The mean age of patients with systemic diseases varied, with diabetes mellitus showing the highest mean age (56.2 ± 8.3 years), followed by hypertension (54.7 ± 7.5 years) and cardiovascular disease (58.4 ± 6.9 years). The prevalence of systemic diseases was significantly associated with endo-perio lesions, with the highest significance found in diabetes mellitus (p < 0.001), hypertension (p = 0.001) and cardiovascular disease (p = 0.022), reflecting their substantial role in the development and progression of these lesions. Conditions such as arthritis, altered kidney function (KFT) and liver dysfunction also showed significant associations, with p-values of 0.008, 0.015, and 0.032, respectively (Table 2). In contrast, the "no disease" group exhibited the lowest mean age of 41.2 ± 6.3 years. Logistic Regression Analysis of Endo-Perio Lesion Severity is described in Table 3. Logistic regression analysis revealed that systemic diseases, especially diabetes mellitus (OR = 2.87), hypertension (OR = 2.45) and altered kidney function (KFT) (OR = 2.13), were significantly predictive of endo-perio lesion severity, with p-values < 0.001, < 0.001 and 0.001, respectively. The odds ratios indicate that these conditions notably increase the likelihood of severe endo-perio lesions. However, liver dysfunction (OR = 1.41, p = 0.102) showed no significant association with lesion severity, suggesting a weaker correlation. Table 4 shows association Between Systemic Disease and Type of Endo-Perio Lesion. The distribution of lesion types varied across different disease groups. For diabetes mellitus, hypertension, and cardiovascular disease, the majority of patients exhibited a balanced spread between primary endodontic and true combined lesions, with diabetes mellitus showing 45% primary endodontic lesions, 25% primary periodontal lesions, and 30% true combined lesions. Hypertension showed 40% primary endodontic lesions and 35% primary periodontal lesions, while cardiovascular disease presented 38% and 32%, respectively. Arthritis showed the highest percentage of primary periodontal lesions (40%) and the lowest proportion of primary endodontic lesions (33%). The no disease group had a higher proportion of primary endodontic lesions (55%) compared to periodontal (25%) and combined lesions (20%). Table 5 indicates age and Gender Distribution of Endo-Perio Lesions with and without Systemic Diseases. Among the age groups, 25-40 years exhibited the highest proportion of participants without systemic diseases (68 male, 37 female), with only 10% of males and 7.9% of females presenting with endo-perio lesions. In contrast, the 56-75 years group had the highest prevalence of endo-perio lesions with systemic diseases, particularly in males (126 out of 155, or 30%) and females (111 out of 138, or 26.4%). Overall, 65.6% (525) of participants had systemic diseases and 34.4% (278) had no systemic diseases. Table 6 indicates correlation of Age Group and gender with type of endo-perio lesion and systemic disease. The 25-40 years age group showed a higher prevalence of primary endodontic lesions in males (36) and females (28), while the 41-55 years group exhibited a higher incidence of true combined lesions, especially among males (52) and females (49). The 56-75 years group had the highest number of true combined lesions in both males (74) and females (72). The total distribution of lesions among participants with systemic diseases included 13.6% with primary endodontic lesions, 24.5% with primary periodontal lesions and 31.5% with true combined lesions.

## Discussion:

This study aimed to assess the demographic distribution, prevalence of systemic diseases and their association with endo-perio lesions in a cohort of 800 participants. Our findings provide valuable insights into the role of systemic conditions in the development and severity of endo-perio lesions, shedding light on the complex interrelationship between oral health and systemic disease. The study cohort showed a slight male predominance, with 52.5% male and 47.5% female participants, a distribution consistent with previous epidemiological studies in oral health [9, 10]. Age-wise, the majority of participants were in the 41-55 years and 56-75 years groups, aligning with the fact that both endodontic and periodontal conditions tend to increase with age, as these lesions are more common in individuals with a longer history of oral hygiene challenges, systemic diseases, and other risk factors (Dwiyanti*et al.* 2023) [10]. The prevalence of systemic diseases in this cohort was notably high, with diabetes mellitus, hypertension and cardiovascular disease showing strong associations with endo-perio lesions. The mean age for diabetes mellitus patients was the highest (56.2 ± 8.3 years), which aligns with known patterns where diabetes increases with age and is a well-established risk factor for both periodontal and endodontic diseases [11, 12-13]. This finding is consistent with existing research indicating that diabetes, hypertension and cardiovascular diseases contribute significantly to the pathophysiology of endo-perio lesions, especially in terms of delayed healing and increased susceptibility to infections (Martínez-García*et al.* 2021) [14]. Furthermore, the significant associations between hypertension (p = 0.001), cardiovascular disease (p = 0.022) and endo-perio lesions highlight the role of systemic vascular health in the oral cavity. These diseases are associated with compromised blood flow to periodontal tissues, thus impairing immune response and making individuals more susceptible to infections that may lead to combined endodontic and periodontal lesions (Löe*et al.* 2021) [15]. Logistic regression analysis confirmed that diabetes mellitus, hypertension, and altered kidney function were significant predictors of the severity of endo-perio lesions. The odds ratios (OR) for diabetes mellitus (OR = 2.87), hypertension (OR = 2.45) and altered kidney function (OR = 2.13) suggest that these conditions substantially increase the likelihood of more severe lesions. This finding is consistent with prior studies where systemic diseases like diabetes and hypertension have been shown to worsen the prognosis of both periodontal and endodontic conditions by impairing the immune response and healing capacity [16, 17]. Interestingly, liver dysfunction showed no significant association (OR = 1.41, p = 0.102), suggesting a weaker or less direct influence on lesion severity. While liver dysfunction may affect overall health and immune function, its impact on oral health, particularly in terms of lesion severity, may be less pronounced than that of more direct systemic conditions such as diabetes or hypertension (Åberg*et al.* 2022) [18]. In the analysis of lesion types, diabetes mellitus, hypertension, and cardiovascular disease displayed relatively balanced distributions between primary endodontic and combined lesions, with a notable prevalence of primary endodontic lesions in the diabetic group (45%). Conversely, arthritis showed a higher proportion of primary periodontal lesions (40%), which could be attributed to the inflammatory nature of both arthritis and periodontal disease, which often coexist [19]. These findings suggest that the systemic diseases might influence the type of lesion seen in endo-perio cases, with some conditions like diabetes predisposing patients to more endodontic issues, while others like arthritis tend to be more closely associated with periodontal problems. The no disease group, which had a significantly lower mean age, exhibited a higher prevalence of primary endodontic lesions (55%). This suggests that younger individuals without systemic diseases may have a higher likelihood of developing isolated endodontic lesions due to factors like trauma, tooth decay, or other local factors that do not involve systemic disease complications [2, 3]. The 56-75 years age group had the highest prevalence of endo-perio lesions, particularly in participants with systemic diseases. This age group showed the highest number of true combined lesions, particularly in males (30%) and females (26.4%). This finding is consistent with the well-documented progression of oral diseases with age, where the cumulative effect of both periodontal and endodontic issues becomes more prominent. The increased prevalence of lesions in individuals with systemic diseases also highlights the compounding effects of aging and systemic health on oral health outcomes [10, 14]. Our data indicate that younger individuals (25-40 years) tend to exhibit a higher prevalence of primary endodontic lesions, while older groups (41-55 years and 56-75 years) are more likely to develop true combined lesions, particularly in the presence of systemic diseases. The increased prevalence of true combined lesions in older individuals aligns with the idea that the burden of systemic diseases and age-related degeneration of both the periodontal and endodontic systems leads to more complex lesions that involve both tissues [14].

## Conclusion:

The role of systemic diseases in the development, severity and type of endo-perio lesions, particularly in older individuals is highlighted. The findings underscore the importance of early diagnosis and management of systemic conditions like diabetes, hypertension and cardiovascular disease in reducing the risk of severe and complex oral lesions. Future research should focus on the mechanisms through which these systemic diseases influence the progression of endo-perio lesions and explore potential therapeutic interventions to mitigate their impact on oral health.

## Figures and Tables

**Table 1:** Demographic distribution of participants (n = 800)

**Variable**	**Frequency (n=800)**	**Percentage (%)**
**Gender**		
Male	420	52.50%
Female	380	47.50%
**Age Group (years)**		
25-40	180	22.50%
41-55	330	41.25%
56-75	290	36.25%

**Table 2:** Prevalence of systemic diseases among patients with endo-perio lesions

**Disease Group**	**Mean Age (Years) ± SD**	**Significance (ANOVA)**
Diabetes Mellitus	56.2 ± 8.3	p < 0.001
Hypertension	54.7 ± 7.5	p = 0.001
Cardiovascular Disease	58.4 ± 6.9	p = 0.022
Arthritis	60.1 ± 7.1	p = 0.008
Liver Dysfunction	52.9 ± 9.4	p = 0.032
Altered Kidney Function (KFT)	55.5 ± 7.8	p = 0.015
No Disease	41.2 ± 6.3	-

**Table 3:** Logistic regression analysis predicting endo-perio lesion severity based on systemic disease

**Predictor**	**Odds Ratio (OR)**	**95% CI**	**p-value**
Diabetes Mellitus	2.87	2.01-4.12	<0.001
Hypertension	2.45	1.66-3.59	<0.001
Cardio Disease	1.98	1.14-3.43	0.014
Arthritis	1.72	1.09-2.72	0.02
Altered KFT	2.13	1.33-3.40	0.001
Liver Dysfunction	1.41	0.91-2.21	0.102

**Table 4:** Association between systemic disease and type of endo-perio lesion

**Disease Group**	**Mean Age (Years) ± SD**	**Primary Endodontic Lesion (%)**	**Primary Periodontal Lesion (%)**	**True Combined Lesion (%)**	**Significance (ANOVA)**
Diabetes Mellitus	56.2 ± 8.3	45%	25%	30%	p < 0.001
Hypertension	54.7 ± 7.5	40%	35%	25%	p = 0.004
Cardiovascular Disease	58.4 ± 6.9	38%	32%	30%	p = 0.022
Arthritis	60.1 ± 7.1	33%	40%	27%	p = 0.005
Liver Dysfunction	52.9 ± 9.4	37%	33%	30%	p = 0.031
Altered Kidney Function (KFT)	55.5 ± 7.8	42%	28%	30%	p = 0.014
No Disease	41.2 ± 6.3	55%	25%	20%	-

**Table 5:** Age and gender distribution of endo-perio lesions with and without systemic diseases (n = 800)

**Age Group (yrs)**	**Gender**	**With Systemic Disease (n, %)**	**Without Systemic Disease (n, %)**	**Total (n)**
25-40	Male	42 (10%)	68 (16.2%)	110
	Female	33 (7.9%)	37 (8.8%)	70
41-55	Male	115 (27.4%)	35 (8.3%)	150
	Female	98 (23.3%)	82 (19.2%)	180
56-75	Male	126 (30%)	29 (6.9%)	155
	Female	111 (26.4%)	27 (6.3%)	138
Total		525 (65.6%)	278 (34.4%)	800

**Table 6:** Correlation of age group and gender with type of endo-perio lesion and systemic disease (n = 800)

**Age Group (yrs)**	**Gender**	**Primary Endo (No Systemic)**	**Primary Perio (With Systemic)**	**True Combined (With Systemic)**	**Total**
25-40	Male	36	16	12	64
	Female	28	14	8	50
41-55	Male	18	52	45	115
	Female	15	49	41	105
56-75	Male	7	34	74	115
	Female	5	31	72	108
Total		109 (13.6%)	196 (24.5%)	252 (31.5%)	557 (with lesions)
